# The bHLH transcription factor CgbHLH001 is a potential interaction partner of CDPK in halophyte *Chenopodium glaucum*

**DOI:** 10.1038/s41598-017-06706-x

**Published:** 2017-08-16

**Authors:** Juan Wang, Gang Cheng, Cui Wang, Zhuanzhuan He, Xinxin Lan, Shiyue Zhang, Haiyan Lan

**Affiliations:** 1 Xinjiang Key Laboratory of Biological Resources and Genetic Engineering, College of Life Science and Technology, Xinjiang University, Urumqi, 830046 China; 2 Institute of Economic Crops, Xinjiang Academy of Agricultural Sciences, Urumqi, 830091 China

## Abstract

Plants have evolved different abilities to adapt to the ever-fluctuating environments for sessility. Calcium-dependent protein kinase (CDPK) is believed to play a pivotal role in abiotic stress signaling. So far, study on the specific substrates that CDPK recognized in response to adversity is limited. In the present study, we revealed a potential interaction between CDPK and a bHLH transcription factor under salt stress in *Chenopodium glaucum*. First, we identified a *CgCDPK*, which was up-regulated under salt and drought stress; then by Y2H screening, CgCDPK was detected to be involved in interaction with a bHLH TF (named as CgbHLH001), which also positively respond to salt and drought stress. Further computational prediction and experiments including GST-pulldown and BiFC assays revealed that potential interaction existed between CgCDPK and CgbHLH001, and they might interact on the plasma membrane. In addition, *CgCDPK*-overexpressed transgenic tobacco line could significantly accumulate transcripts of *NtbHLH* (a homolog of *CgbHLH001* in *N. tabacum*), which provided another evidence of correlation between CgCDPK and CgbHLH001. Our results suggest that CgbHLH001 can interact with CgCDPK in signal transduction pathway in response to abiotic stress, which should provide new evidence for further understanding of the substrate specificity of plant CDPK signaling pathway.

## Introduction

Plants have developed complex networks of signal transduction pathway to adapt to environmental stimuli or avoid damages, i.e. salt overly sensitive (SOS), mitogen-activated protein kinases (MAPK), calcium-dependent protein kinases (CDPK) and abscisic acid (ABA) signaling pathways^1–3^. Among these, CDPK cascades have been suggested to play central roles in response to various abiotic stresses, e.g. cold, drought, salinity, heat and ABA, etc^4–9^. CDPKs are serine/threonine protein kinases that are comprised of four (or five) characterized domains including a variable N-terminal domain, a catalytic domain, a junction domain and a calmodulin-like domain with Ca^2+^-binding EF-hands (C-terminal variable region)^10^. It has been shown that the four distinct domains can define the specific target site of CDPK substrate involved in hormone responses and stress signaling pathways^11–13^.

An increasing evidence in CDPK signaling pathways reveals the interaction between CDPK and its downstream transcription factors (TFs) or proteins in response to stresses^14–16^. Several TFs, including ABF4 (ABA-responsive element-binding factor 4), RSG (repression of shoot growth), Di19–1 (zinc finger) and HsfB2a (heat shock factor B2a) have been characterized as CDPK interaction components *in vivo* which are involved in ABA, salt and drought stress responses^14, 17–19^. Moreover, Arabidopsis CPK10, CPK12 or CPK13 can interact (or phosphorylate) with HSP1 (heat shock protein)^20^, ABI2 (type-2C protein phosphatase)^21^, or KAT1 and KAT2 (K^+^ channel protein)^22^, respectively. However, the comprehensive interactions between CDPK and its substrates have not been well-understood. So far, downstream TFs identified rather than the above mentioned are limited, and which need further study to clarify.

Protein-protein interaction (PPI) plays important roles in signal transduction pathways^23^, many techniques can be employed to analyze such interactions. Among these, three commonly used genetic approaches are yeast two-hybrid system (Y2H)^24^, bi-molecular fluorescence complementation (BiFC)^25, 26^ and glutathione-S-transferse (GST)-pulldown analysis^27^, the former two are *in vivo* while the latter is *in vitro* detection of the interaction. So far, a couple of TFs have been identified as CDPK interaction components via the above technology, e.g. by using ABF4 TF as bait in Arabidopsis, CPK32 is isolated by Y2H screening and shows to modulate the transcriptional function of ABF4^17^. In addition, interactions between AtSLAH3 (S-type anion channel) and AtCPK21, or between AtSLAC1 (another S-type anion channel) and AtCPK23 have been verified by GST-pulldown assay *in vitro* and confirmed by BiFC *in vivo* ^28, 29^. These data suggest that Y2H, GST-pulldown, and BiFC are effective ways in PPI analysis.

*Chenopodium glaucum* is an annual halophyte of Chenopodiaceae and widely distributed in semi-arid area in Xinjiang province, China (Iconographia Cormophytorum Sinicorum Supplementum I, 1983). It is believed that *C. glaucum* has special mechanisms in response to environmental stimuli for its great adaptability to adverse stress while without any special morphological variation^30^. So far, reports on *C. glaucum* are focused on germination and seedling growth under stress, however, the molecular mechanisms of *C. glaucum* in response to stress are not well-understood. Based on the major progress made to uncover the central roles of CDPKs in plant stress signaling networks, we isolated several *CDPK* gene coding sequences from *C. glaucum* and compared their expression in transcriptional level in our previous work. To further explore the role of CDPK in stress signaling pathway, in the present study, by employing a stress response *CDPK* gene as bait, via yeast two-hybrid system, we identified some potential interaction components under salt stress, including two transcription factors (bHLH TF and GATA TF) and other functional proteins. We chose the former TF (shorted as CgbHLH001) for further study, although its functions are becoming widely investigated in plant, no report on such an interaction component of CDPK has been documented so far. Both *in vitro* GST-pulldown and *in vivo* BiFC assays in the present study verified the interaction between CgCDPK and CgbHLH001. In addition, the accumulation of *NtbHLH* (a homolog of *CgbHLH001* in *N. tabacum*) transcripts in *CgCDPK*-overexpressed tobacco transgenic lines supplied another interaction evidence *in vivo*. Our data suggest that CgbHLH001 is a potential interaction partner of CgCDPK in response to salt or drought stress in the signal transduction pathway.

## Results

### Molecular cloning and characterization of *CDPK* from halophyte *C. glaucum*

#### CgCDPK encodes a subgroup II CDPK

**Figure 1:**
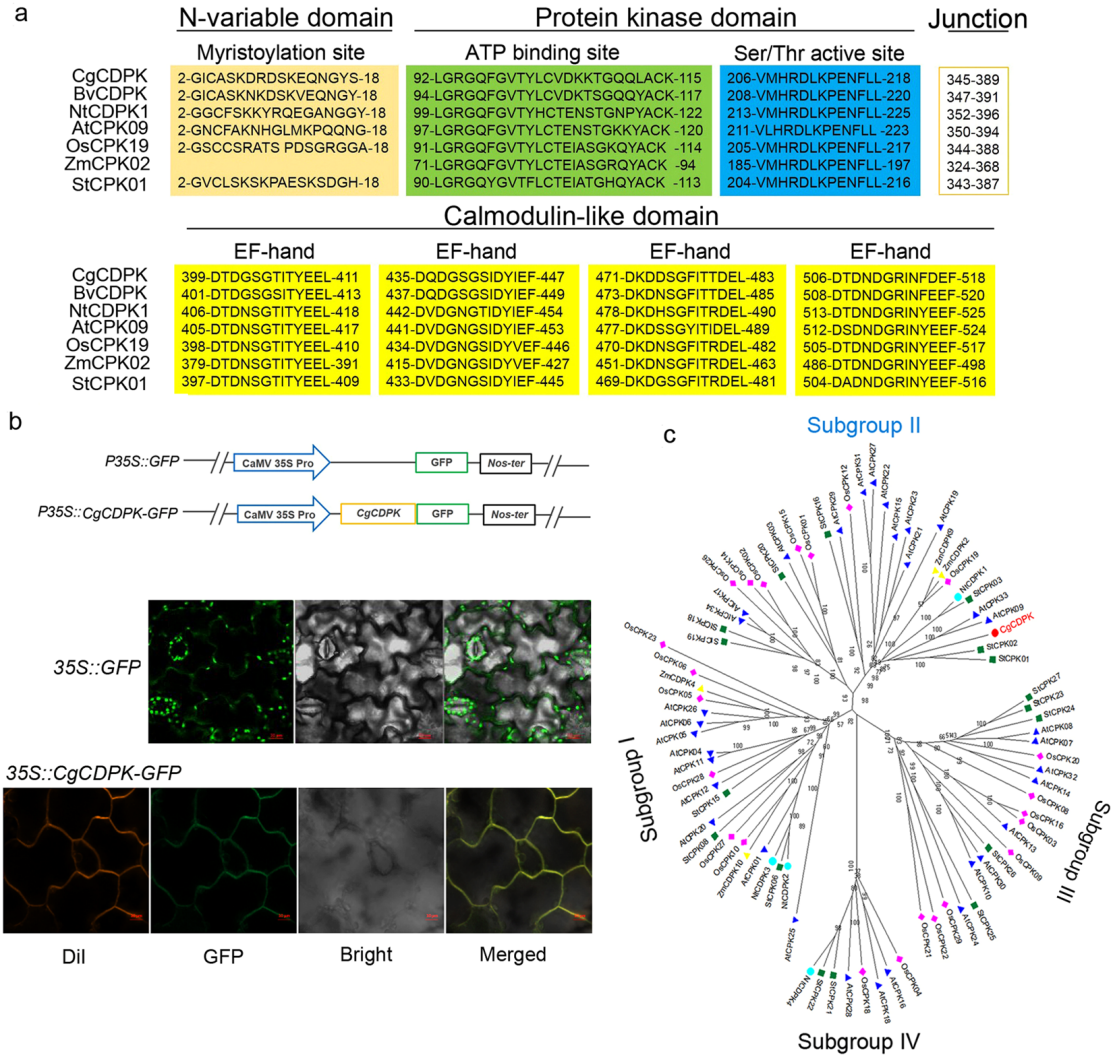
(**a**) Structures of different CDPKs. The N-terminal variable domain contains a myristoylation site (light brown) which is essential for membrane localization, and a protein kinase domain (ATP binding site in green, Ser/Thr active site in blue). Four EF-hands (yellow) within the calmodulin-like domain indicate Ca^2+^ binding motifs. (**b**) Subcellular localization of CgCDPK. Schematic diagrams of constructs of *P35S::CgCDPK-GFP* and *P35S::GFP* (positive control) are at the upper panel; images showed GFP signal of two constructs in transgenic tobacco are at the lower panel. Green fluorescence was observed under the confocal microscope. (**c**) Phylogenetic tree of CDPKs based on the neighbor-joining method. *C. glaucum* CDPK is present in red. Bootstrap analysis was carried out with 1000 replicates. Branches corresponding to partitions reproduced in less than 50% bootstrap replicates are collapsed. Bootstrap values of the branches are indicated in a 0.05 basis. Evolutionary analyses were conducted with MEGA 6.06. At: *Arabidopsis thaliana*; Bv: *Beta vulgaris*; Nt: *Nicotiana tabacum*; St: *Solanum tuberosum*; Os: *Oryza sativa*; Pp: *populus euphratica*; Zm: *Zea mays*.

By RT-PCR method, we isolated a coding sequence (CDS) of *CDPK* gene (shorted as *CgCDPK*) from *C. glaucum*. *CgCDPK* CDS contains a 1 605 bp ORF sequence, which encodes 534 amino acids with a predicted molecular mass of 59.5 kDa and a pI of 6.11. Alignment of the amino acid sequences of CgCDPK and CDPKs from other plants showed that the CgCDPK contains all the four typical domains of CDPK: the N-terminal variable domain, a Ser/Thr kinase domain, an auto-inhibitory junction region and the regulatory calmodulin-like domain (Fig. 1a). In addition, analysis of the amino terminus of CgCDPK with the consensus sequence [Myristoylator (http://us.expasy.org/tools/myristoylator/myristoylator-ref.html)^31^ and NMT Predictor (http://mendel.imp.ac.at/myriatate/SUPLpredictor.htm)^32^] indicates a myristoylation sequence of CgCDPK at 2-GICASKDRDSKEQNGYS-18 region (Fig. 1a), which has been shown to be a signal for plasma membrane localization^33^. Under laser confocal microscope, we observed that red [DiI staining for plasma membrane (PM) marker] and green (*CgCDPK-GFP* overexpression) fluorescent signals were completely merged into yellow color on the PM of the epidermal cells of tobacco leaf (Fig. 1b), it revealed that CgCDPK located on the PM.

Analysis of the evolutionary relationships between CgCDPK and CDPKs from Arabidopsis, rice, maize, tobacco, tomato, etc. showed that CgCDPK belongs to subgroup II (Fig. 1c) out of four subgroups of CDPKs divided previously^34^. CgCDPK shared relatively higher similarity of amino acid sequence to BvCDPK from *Beta vulgaris* (94.12%) and NtCDPK1 (80.13%) from *Nicotiana tabacum*.

#### CgCDPK enhanced the positive response to salt and drought stress

**Figure 2:**
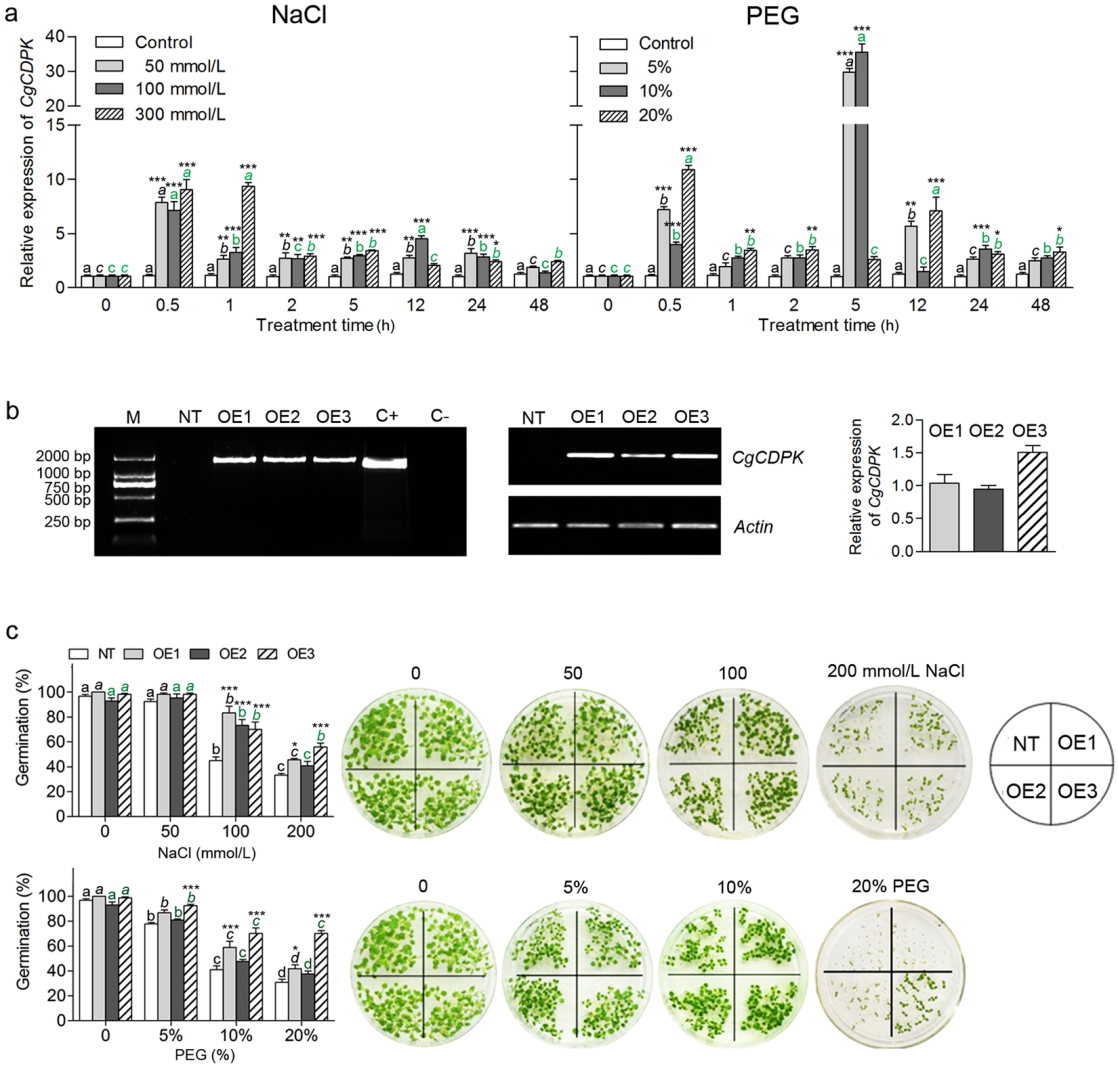
(**a**) Analysis of transcriptional expression pattern of *CDPK* in *C. glaucum* under NaCl and PEG treatment. For the same concentration, different lowercase letters above the columns indicate significant differences (*P* < 0.05) between each different time point and the control; for the same time point, *,**, and *** are used to indicate significant difference existing between each different concentration and the control at 0.05, 0.01, and 0.001 levels. Values are means ± SE of six replicates. (**b**) PCR, RT-PCR and qPCR analyses of *CgCDPK* transgenic tobacco lines. Left: PCR; middle: RT-PCR; right: qPCR. M: DL2000 DNA marker; NT: Non-transgenic tobacco plant. Lane 2–4: Transgenic tobacco lines (OE1, OE2, OE3); C+: Positive control; C-: Negative control. (**c**) Germination percentage of NT and T1 OE lines on MS medium containing different concentrations of NaCl and PEG6000. For the same OE line, different lowercase letters above the columns indicate significant difference (*P* < 0.05) between the different concentration and the control; for the same concentration, *,**, and *** are used to indicate significant difference existing between each different OE line and NT tobacco plant at 0.05, 0.01, and 0.001 levels. Values are means ± SE of four replicates.

After being exposed to varying concentrations of NaCl and PEG, *C. glaucum* plant was analyzed with the response of *CgCDPK* at transcriptional level (Fig. 2a). It was found that salt stress significantly enhanced the accumulation of *CgCDPK* transcripts shortly after treatment, it was about 8-fold greater at 0.5 h under different NaCl concentrations than that of the control (*P* < 0.001), after then (from 1 h to 48 h) which fell down sharply (except for 300 mmol/L at 1 h), but still significantly higher than the control (Fig. 2a; left). For PEG treatment, the transcriptional level of *CgCDPK* increased much higher at 0.5 h for all PEG concentrations (*P* < 0.0001) and then fell down, which was similar to that of the NaCl treatment within a short time; however, the expression of *CgCDPK* rose again and reached to the maximum (about 30-fold more than control) at 5 h (except for 20%) (*P* < 0.0001) (Fig. 2a; right), which was different with that of the NaCl stress. In order to further explore the function of CgCDPK in response to abiotic stress, we examined seed germination behavior of *CgCDPK*-overexpressed transgenic tobacco lines under salt and drought stress. Results showed that transgenic lines were less sensitive to NaCl or PEG in germination than NT plants, especially OE1 and OE3 at higher concentrations (Fig. 2b,c). In combination of these results, it suggests that *CgCDPK* gene can positively and quickly respond to abiotic stress.

### Screening and verification of CDPK interaction components by yeast two-hybrid system

#### Construction of cDNA library in *C. glaucum* under salt stress

Total RNA was isolated from leaves of *C. glaucum* under 300 mmol/L NaCl treatment with the ratio of A_260_/A_280_ = 2.05, and mRNA was properly purified with a smear distributed between 500 bp and 5000 bp. Then the cDNA library was constructed with a total colony-forming unit (CFU) as 1.26 × 10^7^, which meets the requirement of cDNA library construction. Twenty singular colonies from one plate were randomly selected to check the insert fragment distribution by PCR test, which suggests a 100% recombinant frequency, and the fragment size was distributed from 850 bp to 2000 bp (Supplementary Fig. 1).

#### Screening and verification of CDPK interaction components by split-ubiquitin based membrane yeast two-hybrid analysis

**Figure 3:**
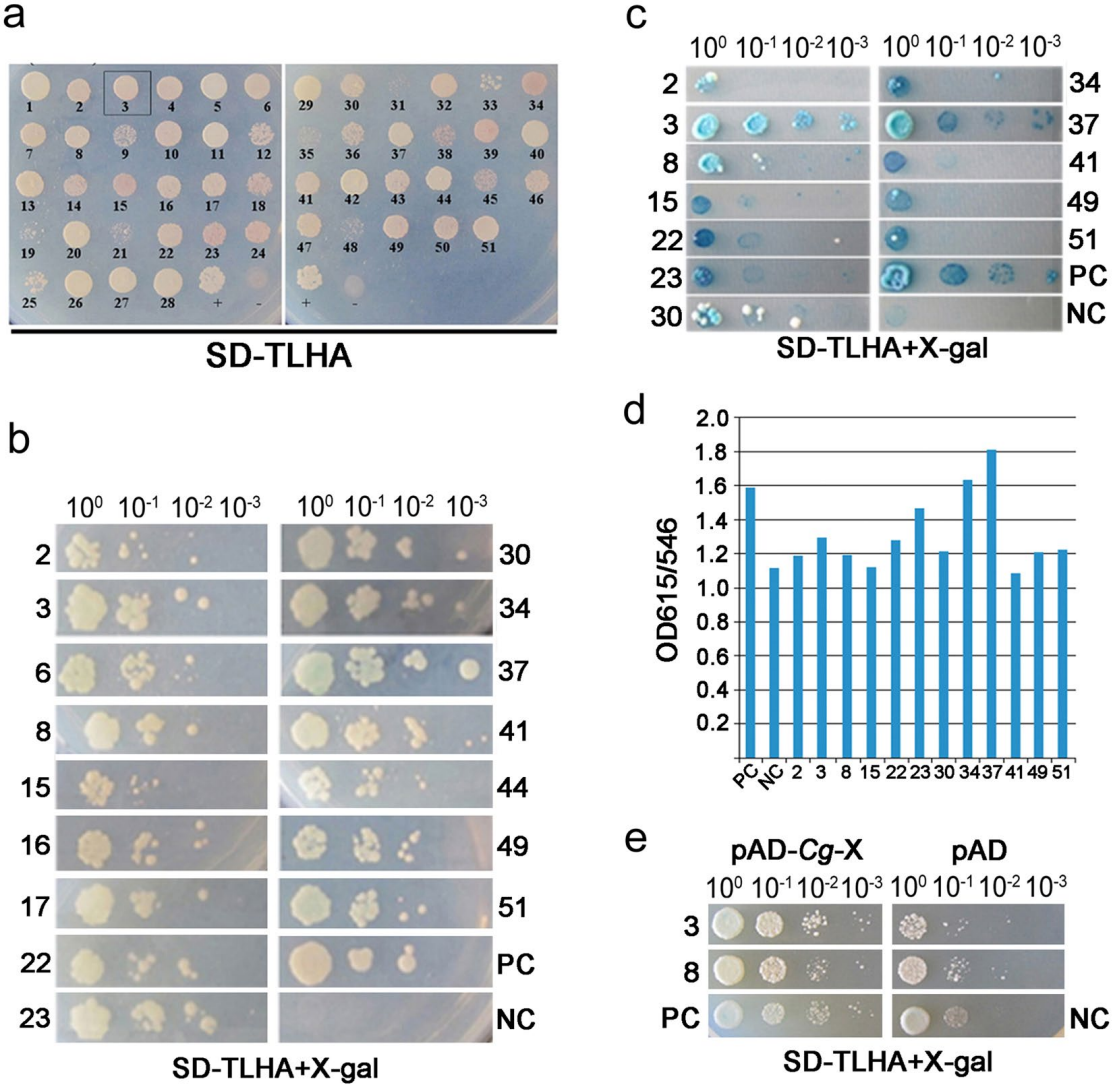
Screening and verification of CDPK interaction components of *C. glaucum* by split-ubiquitin based membrane yeast two-hybrid analysis. (**a**) Initial screening using CDPK as bait; (**b**,**c**) the first and second verification by back-transformation of the positive clones from cDNA library; (**d**) the activity of β-galactosidase (reporter gene *Lac Z*) of the positive clones confirmed by the second back-transformation. (**e**) Self-activation detection of CDPK interaction components. pBD-*CgCDPK*: yeast expression vector of bait gene-*CgCDPK*; pAD: yeast prey vector as negative control; pAD-*Cg*-*X*: yeast expression vector of prey cDNA derived from the cDNA library of *C. glaucum* under salt stress; SD-TLHA + X-gal: Trptophan, Leucine, Histidine, Adenine synthetic dropout basic yeast culture medium with addition of X-gal. PC: Positive control, pTSU2-APP and pNubG-Fe65 co-transformed yeast strain; NC: negative control, pTSU2-APP and pPR3N co-transformed yeast strain.

To identify the components that potentially interact with CgCDPK, we performed a split-ubiquitin based membrane yeast two-hybrid screening of a cDNA library of *C. glaucum* using CDPK as bait and obtained 51 clones that encode 32 potential candidate proteins (Fig. 3a), which were classified as transcription factors (TFs) and proteins with predicted functions in protein synthesis, photosynthetic pathway, stress tolerance and metabolism.

Subsequently, the positive clones were further screened by the reporter gene - *His, Ade* expression on SD/-TLHA medium for the first (Fig. 3b) and the second (Fig. 3c) verification; meantime, another reporter gene *LacZ* expression was detected by measuring β-galactosidase activity (Fig. 3d), finally we got 12 candidate clones which showed potential interaction with CgCDPK. Self-activation detection revealed that two candidate clones showed similar reaction with negative control (Fig. 3e). Based on above analysis, we chose No.3 clone which encodes a bHLH transcription factor (named as CgbHLH001) for the following study.

### Characterization of CDPK interaction component-CgbHLH001 in *C. glaucum*

#### Molecular identification of CgbHLH001 from *C. glaucum*

**Figure 4:**
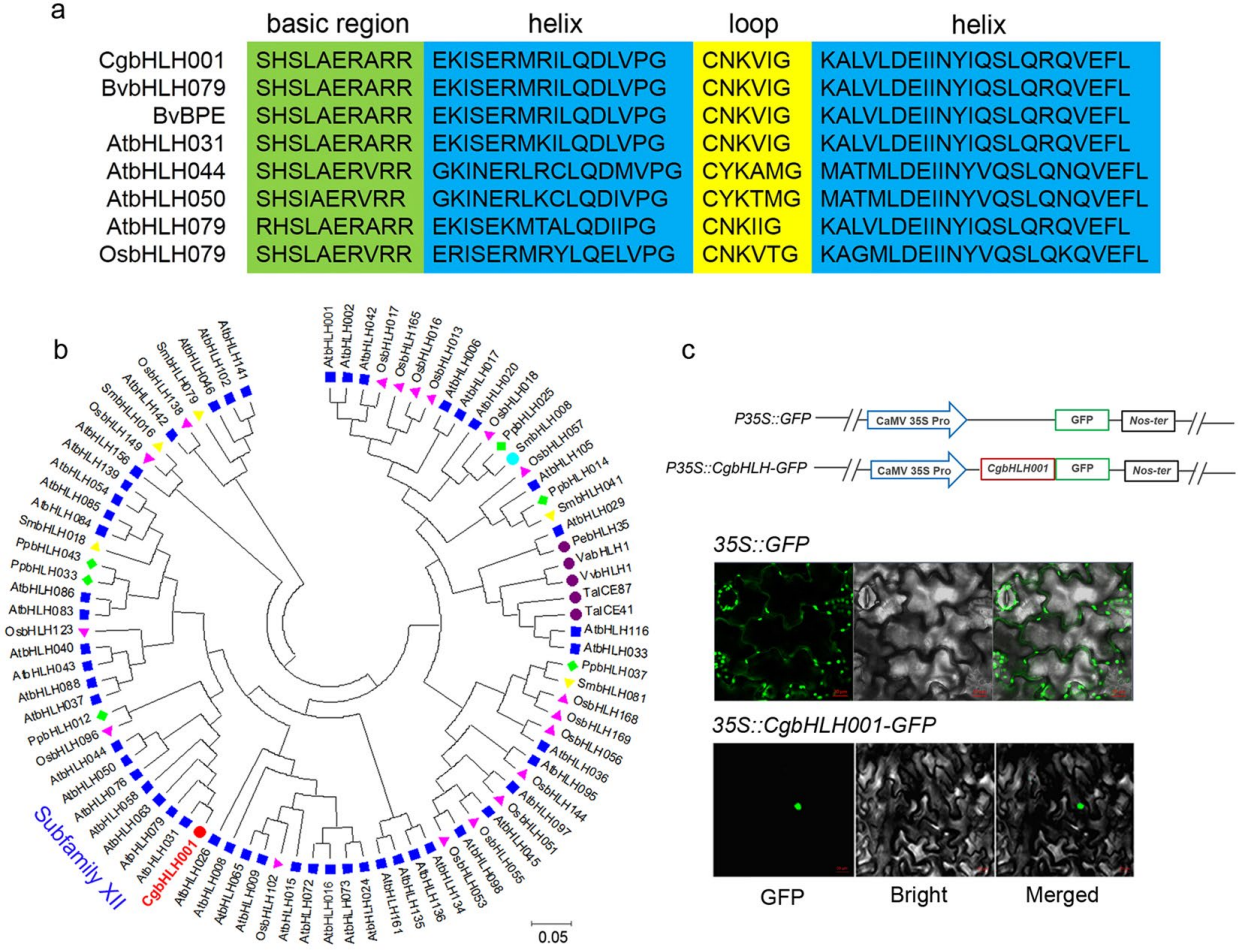
(**a**) Alignment of bHLH domains of representative plant species using DNAMAN 8.0 software. Seven representative genes from bHLH subfamily XII are shown. Different color sections indicate the position of DNA-binding basic region (green), two α-helices (blue), and the variable loop region (yellow). (**b**) Phylogenetic analysis of amino acid sequences of various plant bHLH based on the neighbor-joining method. Accession numbers are indicated after species names from GenBank. *C. glaucum* bHLH transcription factor (CgbHLH001) is present in bold and red background. Bootstrap analysis was carried out with 1000 replicates. Branches corresponding to partitions reproduced in less than 50% bootstrap replicates are collapsed. Bootstrap values of the branches are indicated in a 0.02 basis. Evolutionary analyses were conducted in MEGA 6.0. (**c**) Subcellular localization of CgbHLH001. Schematic diagrams of constructs of *P35S::CgbHLH001-GFP* and *P35S::GFP* (positive control) are at the upper panel, images showed GFP signal of two constructs in transgenic tobacco are at the lower panel. Green fluorescence was observed under the confocal microscope. Va: *Vitis amurensis*; Vv: *Vitis vinifera*; Ta: *Triticum aestivum*.

Full length cDNA of bHLH transcription factor-*CgbHLH001* of *C. glaucum* was isolated from cDNA library constructed earlier. *CgbHLH001* contains 792 bp and encodes 263 amino acid residues with a predicted molecular weight of 28.60 kDa and pI of 6.80. Structural analysis (http://www.ebi.ac.uk/interpro/; Iterative Threading ASSEmbly Refinement)^35^ suggests that the secondary structure of CgbHLH001 presents a typical MYC-type bHLH domain (IPR011598) and a coiled coil region (Fig. 4a). Furthermore, phylogenetic analysis suggests that plant bHLH proteins are monophyletic and constitute 26 subfamilies^36^. Seven well-characterized bHLHs in subfamily XII are listed in Fig. 4b, in which CgbHLH001 showed the highest homology to BvbHLH79, BvBPE of *Beta vulgaris subsp. vulgaris*, and to AtbHLH031 and AtbHLH079 of *Arabidopsis thaliana*. In addition, the amino acid sequence revealed a putative nuclear localization signal (NLS) within bHLH domain of CgbHLH001. Further verification by transgenic tobacco of *P35S::CgbHLH001-GFP* showed that strong green fluorescence was exclusively localized to the nucleus, which may support a role for CgbHLH001 as nuclear transcription factor (Fig. 4c).

#### *CgbHLH001* expression is induced by abiotic stress

**Figure 5:**
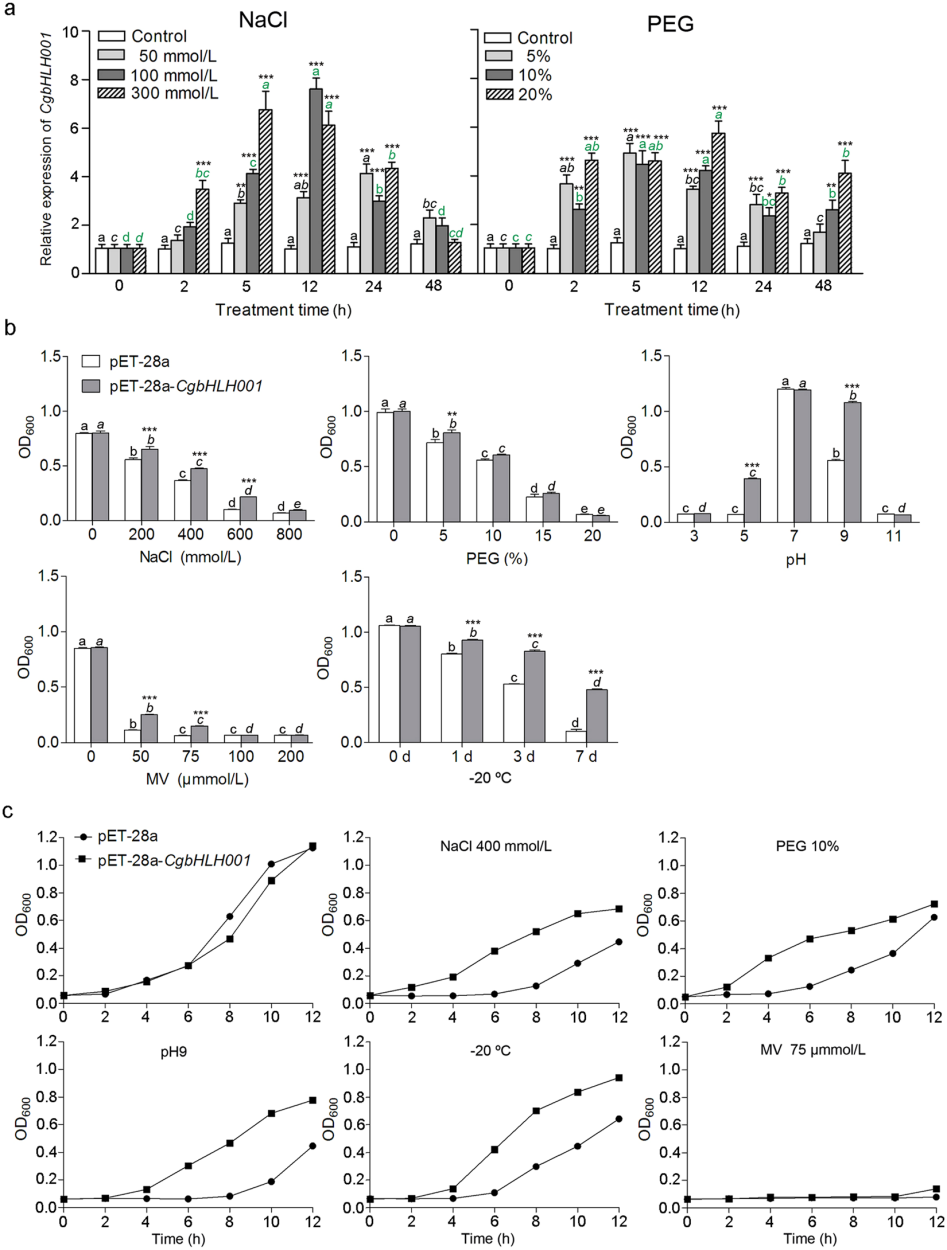
(**a**) Analysis of transcriptional expression pattern of *CgbHLH001* in *C. glaucum* under NaCl and PEG treatment. For the same concentration, different lowercase letters above the columns indicate significant differences (*P* < 0.05) between each different time point and the control; for the same time point, *,**, and *** are used to indicate significant difference existing between each different concentration and the control at 0.05, 0.01, and 0.001 levels. Values are means ± SE of six replicates. (**b**) Growth measurement of recombinant strain (Transetta: pET-28a-*CgbHLH001*) under various abiotic stresses. The culture was harvested at 12 h. For the same strain, different lowercase letters above the columns indicate significant difference (*P* < 0.05) between each treatment and the control; for the same treatment, *,**, and *** are used to indicate significant difference existing between the recombinant strain and the control strain. (**c**) Time course of growth of the recombinant strain (Transetta: pET-28a-*CgbHLH001*) under different abiotic stresses. The culture (200 µL) was sampled at every 2 h till a total of 12 h. In (**b**) and (**c**): Transetta: pET-28a was used as control. Values are means ± SE of three replicates.

Transcriptional expression of *CgbHLH001* in *C. glaucum* was analyzed after exposure to salt and drought stress. Under NaCl or PEG treatment, *CgbHLH001* was upregulated with time increasing, the highest expression level was from 5 h to 12 h (for NaCl or PEG, *P* < 0.0001) (Fig. 5a), the higher the concentration was, the earlier the highest expression value was presented. *CgbHLH001* shared similar transcriptional pattern under NaCl and PEG treatments, but its response to the former was much greater than that of the latter. These data indicate that *CgbHLH001* can positively respond to salt or drought stress. To make further understanding of gene function in response to stress, the ectopic expression of *CgbHLH001* in *E. coli* was analyzed. The growth performance of *E. coli* strain harboring with recombinant plasmid pET-28a-*CgbHLH001* was examined under various stresses, i.e. different concentrations of NaCl, PEG, methyl viologen, different range of pH, and low temperature (−20 °C). Compared to the control strain, the recombinant strain could tolerate much broader range of different stresses (Fig. 5b) and grow much better (Fig. 5c), especially under 400 mmol/L NaCl, pH 9 and −20 °C treatment. The above data indicate that *CgbHLH001* overexpression in prokaryote is able to improve the stress tolerance of the recombinant strain.

### Analysis of interaction between CDPK and bHLH in *C. glaucum*

#### Prediction of interaction between CgCDPK and CgbHLH001

**Figure 6:**
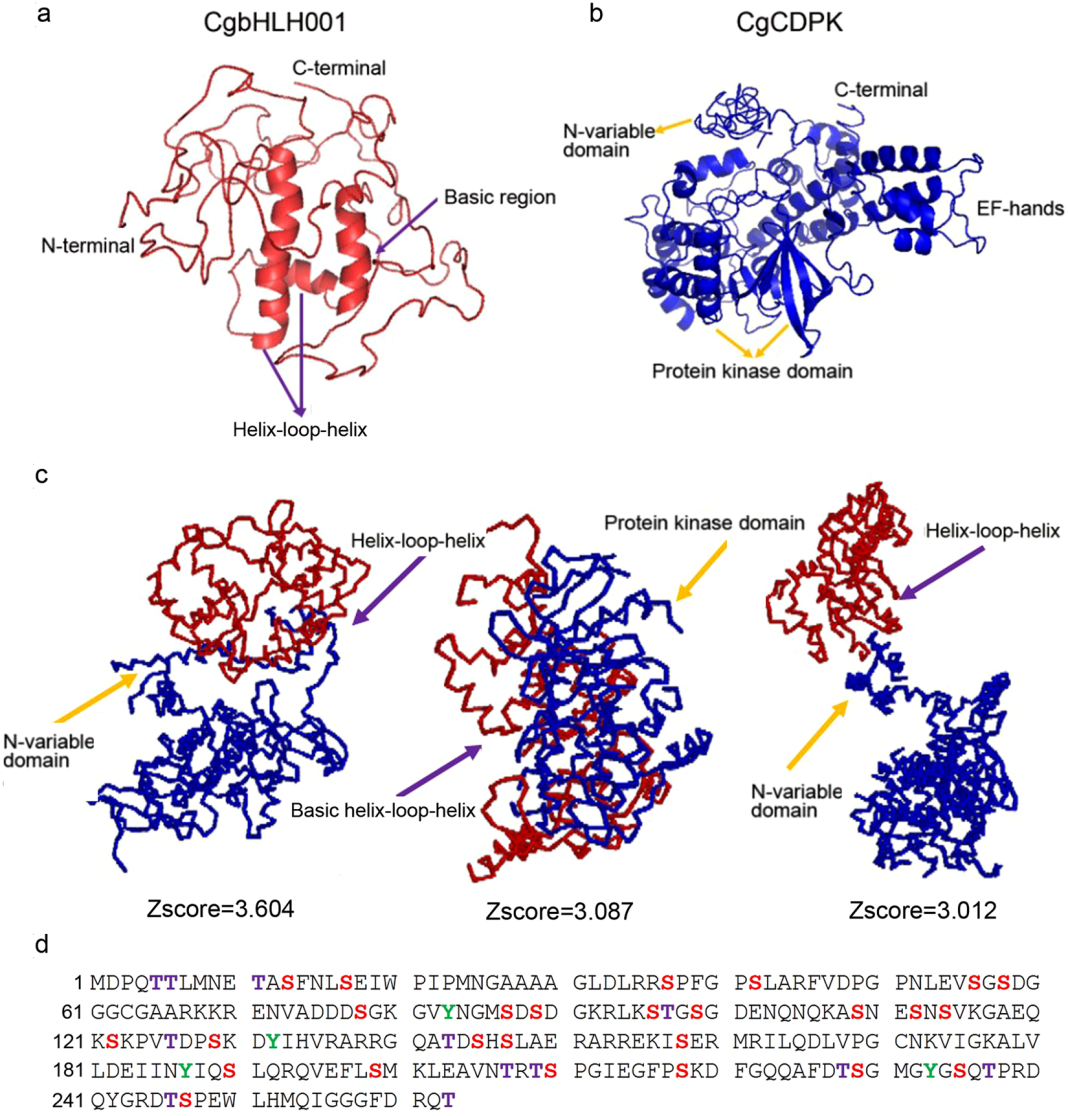
Predicted models of the interaction between CgCDPK and CgbHLH001. (**a**,**b**) Model of CgbHLH001 or CgCDPK by I-TASSER software. (**c**) Three top ranking models of the interaction complex by Prosite and a new dimeric threading algorithm (COTH)^48^. Red part represents CgbHLH001, blue part is CgCDPK. (**d**) Phosphorylation sites of CgbHLH001 predicted by Kinase Phos 2.0 program (http://kinasephos2.mbc.nctu.edu.tw/publication.html). Amino acids in color indicate possible phosphorylation sites.

Prediction of 3D structure of CgCDPK, CgbHLH001 and the probable complexes of CgCDPK-CgbHLH001 interaction based on the sequences revealed different models in Fig. 6, which showed that CgbHLH001 had a typical basic helix-loop-helix conserved domain (Fig. 6a), and CgCDPK had the structure with an N-terminal region, a protein kinase domain and several EF-hand domains (Fig. 6b). The three top ranking models of the CgCDPK-CgbHLH001 interaction complex were presented in Fig. 5c, which indicate the possible interaction sites between helix-loop-helix domain of CgbHLH001 and protein kinase domain or the N-variable terminal domain of CgCDPK (Fig. 6c). Prediction of phosphorylation sites of CgbHLH001 revealed 39 potential amino acid residues (Fig. 6d), in which the motif of ^61^-GKRLKS-^66^ was a putative action site for CgCDPK.

#### GST-pulldown verification of protein interaction *in vitro*

**Figure 7:**
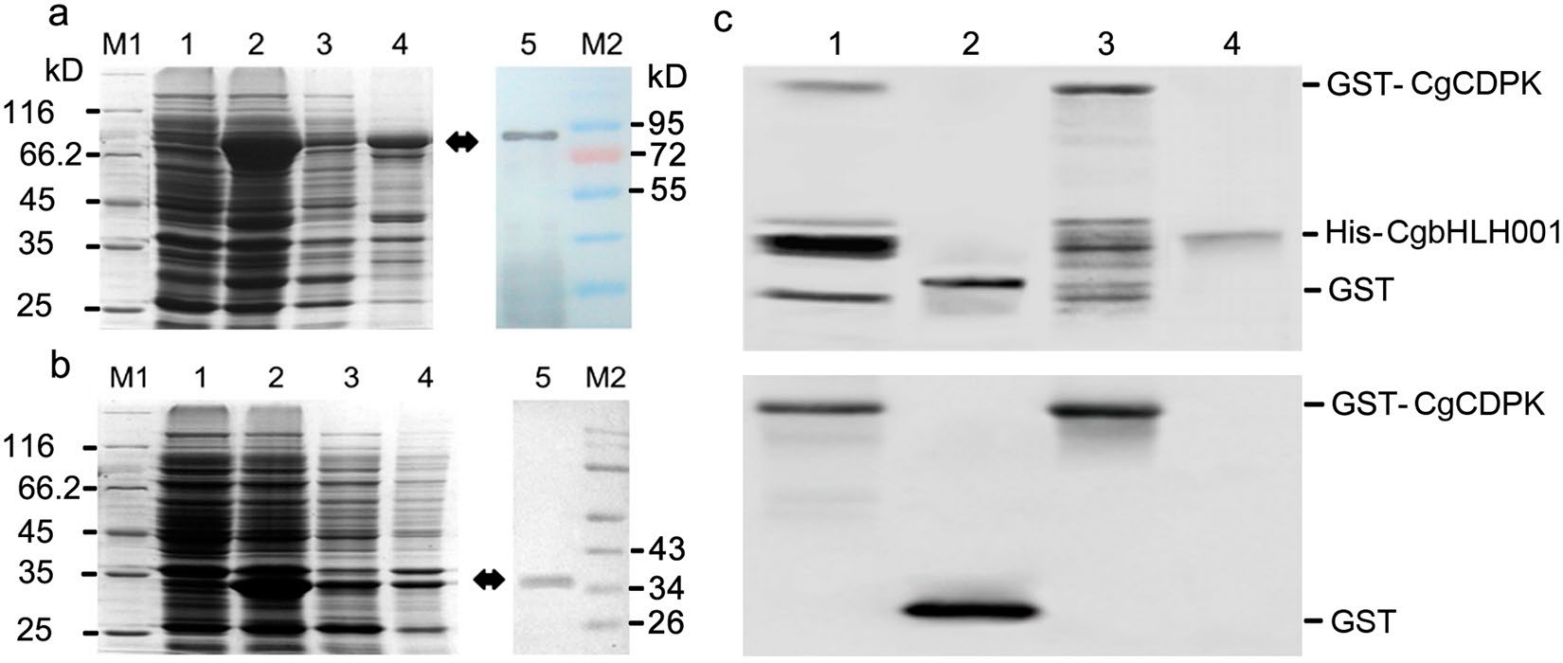
Validation of interaction between CgCDPK and CgbHLH001 *in vitro*. (**a**) SDS-PAGE analysis of total protein of pGEX-4T-1-*CgCDPK* and detection of CDPK by Western blot. Molecular weight (MW) of GST-CgCDPK is about 85 kDa; (**b**) SDS-PAGE analysis of total protein of pET-28a-*CgbHLH001* and detection of CgbHLH001 by Western blot. MW of His-CgbHLH001 is 34 kDa; M1, M2: protein MW marker; 1: before induction; 2: after induction; 3: supernatant; 4: precipitation. Arrow indicates GST-CDPK protein band in (**a**) or His-CgbHLH001 protein band in (**b**,**c**) GST**-**pulldown assay. Ni-His-CgbHLH001 resin was incubated with purified GST-CgCDPK and eluted with imidazole, then resolved by SDS-PAGE and detected by Western blot. Upper panel: Coomassie brilliant blue staining; lower panel: Anti-GST Western blot assay. Lane 1: Pulldown result of His-CgbHLH001 and GST-CgCDPK; lane 2: purified GST; lane 3: purified GST-CgCDPK and lane 4: purified His-CgbHLHH001.

In the present study, His-tagged CgbHLH001 was immobilized on Ni column and assayed for the ability to pull down the GST-CgCDPK fusion protein (Fig. 7a,b). Pulldown results were analyzed by immunoblotting with anti-GST antibody (Fig. 7c). As shown in the first lane of Fig. 7c, which presented the pulldown result compared to the GST only (lane 2), purified GST-CDPK (lane 3) and purified His-CgbHLH001 (lane 4), both His-CgbHLH001 and GST-CgCDPK bands were simultaneously present in Coomassie blue staining gel (Fig. 7c; upper panel), further western blot analysis detected GST-CgCDPK existence (Fig. 7c; lower panel), which suggests that His-CgbHLH001 can interact with and pull down GST-CDPK *in vitro*.

#### BiFC assay detects protein interaction *in vivo*

**Figure 8:**
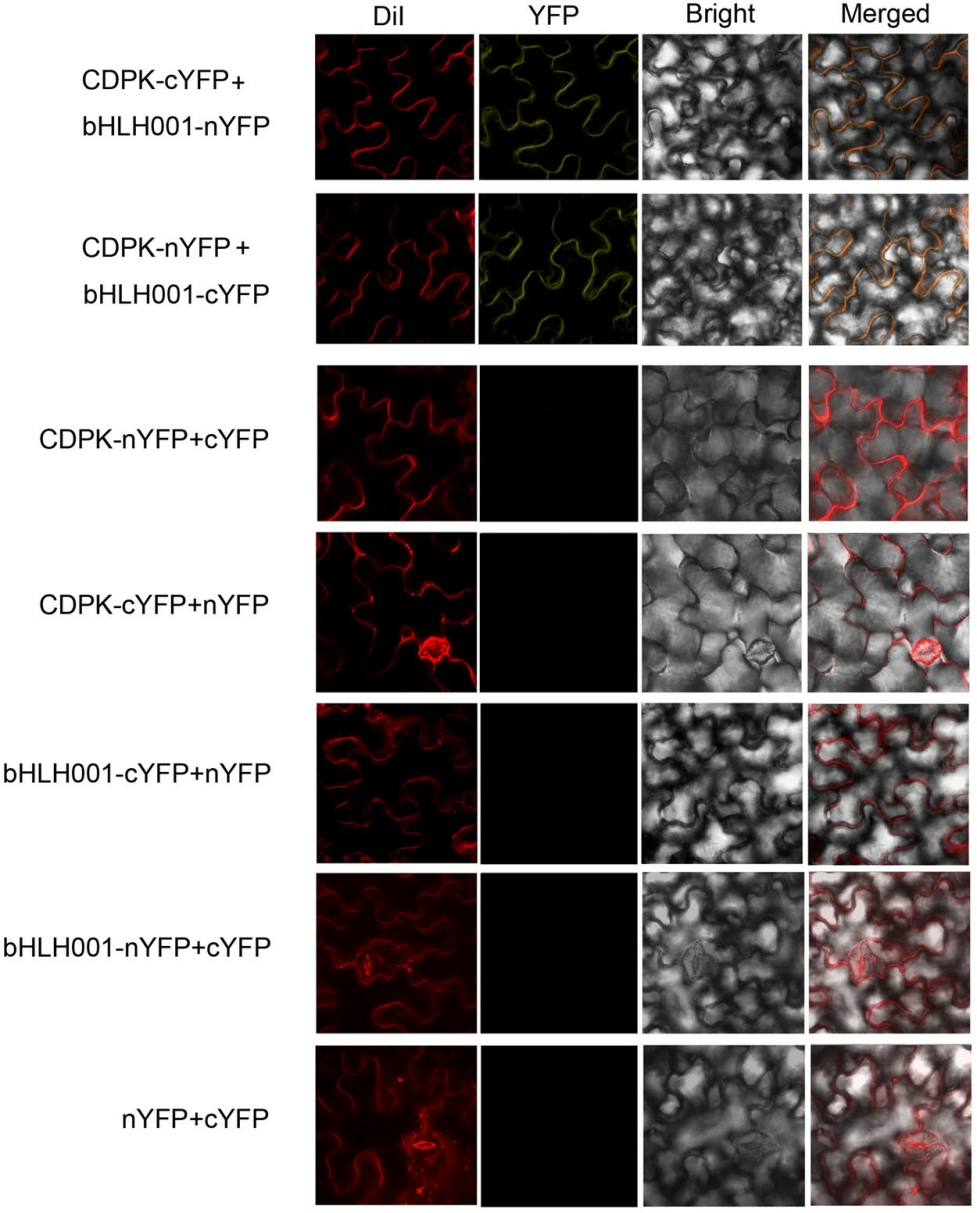
BiFC analysis of interaction between CgCDPK and CgbHLH001 *in vivo*. The C-terminal half and the N-terminal half of YFP were fused to CgCDPK (CgCDPK-cYFP and CgCDPK-nYFP) and CgbHLH001 (CgbHLH001-cYFP and CgbHLH001-nYFP). YFP fluorescence was detected in *N. benthamiana* fresh leaves co-infiltrated with combinations of CgCDPK-cYFP + CgbHLH001-nYFP and CgCDPK-nYFP + CgbHLH001-cYFP. Combinations between any above fused construct and non-fused cYFP or nYFP, as well as cYFP + nYFP were used as controls. The plasma membrane was marked by DiI staining. Fluorescence was visualized by confocal microscope. The experiment was repeated two times with similar results. Bars = 50 µm.

To further characterize interaction between CgCDPK and CgbHLH001 *in vivo*, bimolecular fluorescence complementation assay was performed by co-infiltration of recombinant strain combination − *35S::CgCDPK-nYFP* + *35S::CgbHLH001-cYFP* or *35S::CgCDPK-cYFP* + *35S::CgbHLH001-nYFP* into *N. benthamiana* fresh leaf to observe the generation of fluorescence. As a result, a strong yellow fluorescent signal was observed on the PM of the epidermal cells when either of the above combination was delivered into the tobacco plant, compared to no fluorescent signal in cells with any of other combinations (Fig. 8). Meantime, the tobacco leaves were simultaneously treated with a PM fluorescent dye, which further verified that CgbHLH001 potentially interacted with CgCDPK on the PM.

#### CDPK may regulate downstream transcription factor bHLH

**Figure 9:**
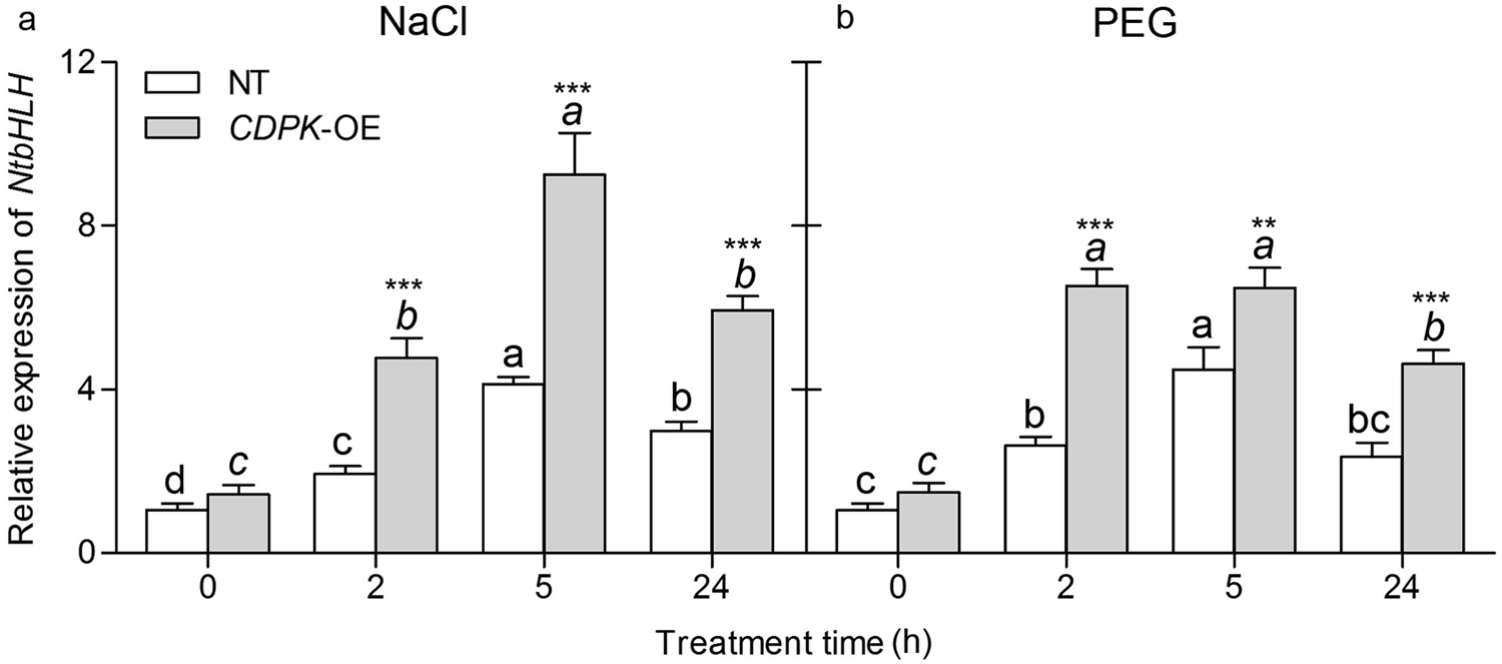
Analysis of transcript accumulation of *NtbHLH* in *CgCDPK*-overexpressed transgenic tobacco line under stress. (**a**) 200 mmol/L NaCl treatment; (**b**) 15% PEG6000 treatment. For the same line, different lowercase letters above the column indicate significant difference (*P* < 0.05) between the different time point and the control; for the same time point, *,**, and *** are used to indicate significant difference existing between the transgenic line and NT plant at 0.05, 0.01, and 0.001 levels. Values are means ± SE of six replicates. *CDPK-*OE: *CDPK*-overexpressed tobacco transgenic line; NT: non-transgenic plant; *NtbHLH*: a homolog of *CgbHLH001* gene in tobacco.

To confirm the interaction between CgCDPK and CgbHLH001 *in vivo*, transgenic tobacco lines with *CgCDPK* overexpression were generated and the transcriptional level of *NtbHLH* (a homolog of CgbHLH001 in *N. tabacum*) was analyzed by quantitative RT-PCR. Results showed that the transcripts of *NtbHLH* was significantly accumulated under NaCl or PEG treatment from 2 h to 24 h tested, the highest expression level was observed around 2–5 h with more than 2.0-fold in *CgCDPK*-overexpressed line than that of the NT plant (for NaCl or PEG, *P* < 0.0001) (Fig. 9a,b), which suggests that *CgCDPK* overexpression can induce expression of *NtbHLH -* a homolog of *CgbHLH001* in *N. tabacum* under salt or drought stress.

## Discussion

Calcium-dependent protein kinases (CDPKs) are key stress sensors and signal transducers of calcium signaling pathway in plants, which play important roles in response to environmental stimuli^37^. Identification of the interaction components is an efficient way to reveal CDPK functions^38^. So far, limited reports on the interaction between CDPK and its substrates have been documented. In the present study, we identified an interaction component of CDPK - a basic helix-loop-helix (bHLH) transcription factor (TF) from *C. glaucum* by yeast two hybrid (Y2H) screening, further *in vitro* pulldown and *in vivo* BiFC assay as well as transgenic plant verification all provided evidence that CgCDPK and CgbHLH001 could positively respond to abiotic stress and potentially interact in transduction of signal to enhance stress tolerance.

In the present study, a subfamily II CgCDPK was identified from *C. glaucum* and was observed to be localized on the PM, it is consistent with the prediction that a myristoylation (with a Gly residue at position 2) and a palmitoylation (with a Cys residue at position 4) sites are harbored in the N-terminal sequence - M**G**I**C**ASKDRDSKEQNGYS of CgCDPK, which is associated to membrane localization^39, 40^. It has been reported that protein myristoylation can also promote protein-membrane or protein-protein interaction, which may occur co-translationally and is irreversible^41, 42^; afterwards the reversible palmitoylation of the protein may further stabilize or regulate the interaction and enable CDPK to shuttle between membrane and the cytosol or nucleus in response to stress signals^40, 43^, which has experimentally been verified with some CDPKs in Arabidopsis, rice, and other flowering plant species^43, 44^.

CDPKs have long been considered to be involved in various abiotic stresses, e.g. AtCPK3, AtCPK4, AtCPK11, AtCPK23, AtCPK27 (subfamily II) in Arabidopsis^4, 16, 45^. By contrast, AtCPK12, the closest homolog of AtCPK4/AtCPK11, plays the opposite role in stress tolerance^21^, which suggests that the commitment of the CDPK functions may depend on the downstream interaction components. In the present study, the positive responses of *CgCDPK* to salt and drought in *C. glaucum* were in line with AtCPK4 and AtCPK11 in Arabidopsis, and ZmCPK4 in maize^18, 46^. The quick activation of CgCDPK transcription under NaCl and PEG treatment may suggest that this kind of kinase in the upstream responds stress earlier. Compared to the reported transcriptional level of *ZmCPK4* after 3 h PEG treatment, that of *CgCDPK* at 5 h in the present study was a much greater increase, the details still remain to be explained by further experiments. In addition to the active response of *CgCDPK* to abiotic stress, seed germination of *CgCDPK*-overexpressed transgenic tobacco line was much less sensitive to salt and drought stress. All these data indicate that CgCDPK should be important in stress tolerance.

CDPKs are major signaling molecules that are involved in a variety of stress responses, however, the molecular mechanisms in the interaction between CDPK and the specific substrate are still largely unknown^11^. So far, a limited number of transcription factors or proteins have been characterized as the CDPK interaction components, e.g. StCDPK5 (*Solanum tuberosum*) and NtCDPK1 (*Nicotiana tabacum*), two homologs of CgCDPK in the present study, are dominantly localized to the PM, the former can activate StRBOHB (an NADPH oxidase) on the PM by phosphorylating the N-terminal region^14^; the N-variable domain of NtCDPK1 can interact with a bZIP TF-RSG (repression of shoot growth) in a Ca^2+^-dependent manner and specifically phosphorylates Ser^114^ of RSG^11, 47^. In the present study, we identified a potential substrate of CgCDPK, a bHLH TF - CgbHLH001, by Y2H screening, GST-pulldown and BiFC assay. The predicted interaction complexes revealed that the N-variable domain of CgCDPK could interact with CgbHLH001^48^. A phosphorylation site in ^61^-GKRLKS-^66^ (Ø-X-R/K-X-X-S, Ø is a hydrophobic residue and X is any residue)^49^ out of thirty-nine predicted ones of CgbHLH001 is likely to be the specific action site of CDPK, which agreed with the predicted interaction pattern at N-variable domain. A tobacco CDPK1 (located on the PM)^47^ was reported to interact with and phosphorylate RSG -a leucine zipper transcription factor, finally make it translocate from cytoplasm to nucleus and regulate the target gene expression^50^. In combination with the PM localization of CgCDPK and the potential interaction between CgbHLH001 and CgCDPK on the PM revealed by BiFC assay in the present study, we speculate that these two components should interact on the PM before CgbHLH001 can make further actions. In addition, our investigation with the transgenic tobacco line showed that overexpression of *CgCDPK* significantly accumulated the transcripts of *NtbHLH* (a homolog of *CgbHLH001* in *N. tabacum*) under stress conditions, which may provide further evidence *in vivo* for the relationship existed between CgCDPK and CgbHLH001. So far, few reports have been documented on bHLH TF as the substrate of plant CDPKs. Our findings on the interaction between CgbHLH001 and CgCDPK should be an important contribution to this field.

bHLH proteins are the second largest family of plant TFs which are classified into 26 subgroups by phylogenetic analysis^36^. They can function as activators of one set of genes and repressors of others^51^. In Arabidopsis or rice genome, 162 or 167 bHLH proteins have been identified, and about 30% of which have been functionally characterized, the role includes regulatory networks of plant growth, development and stress responses^52, 53^, however, only a few members have been shown to be involved in stress tolerance. In the present study, phylogenetic analysis revealed that CgbHLH001 belongs to typical XII subgroup of bHLH TF family, and the accumulation of *CgbHLH001* transcripts under salt or drought stress was consistent with the performance of *bHLH* genes observed in Arabidopsis, rice, soybean, etc^54, 55^. The best-studied bHLH TFs are members of subgroup III, which are largely involved in responses to abiotic stress, e.g. *AtMYC2*, *AtbHLH17*, *AtbHLH92* and *AtICE1* in Arabidopsis, *OrbHLH001*, *OrbHLH002* in wild rice were shown to be upregulated by drought, salinity or cold stress^56–61^. So far, however, much limited reports on bHLH TF in XII subgroup have been recorded in stress tolerance. Our results indicate that ectopic expression of *CgbHLH001* could confer *E. coli* strain with enhanced stress tolerance, which means that helix-loop-helix in CgbHLH001 may have similar function with helix-turn-helix in prokaryote in regulation of relevant gene expression^62^. Our preliminary investigation on CgbHLH001 may provide an evidence for understanding the role of bHLH TFs in XII subgroup in stress responses.

**Figure 10:**
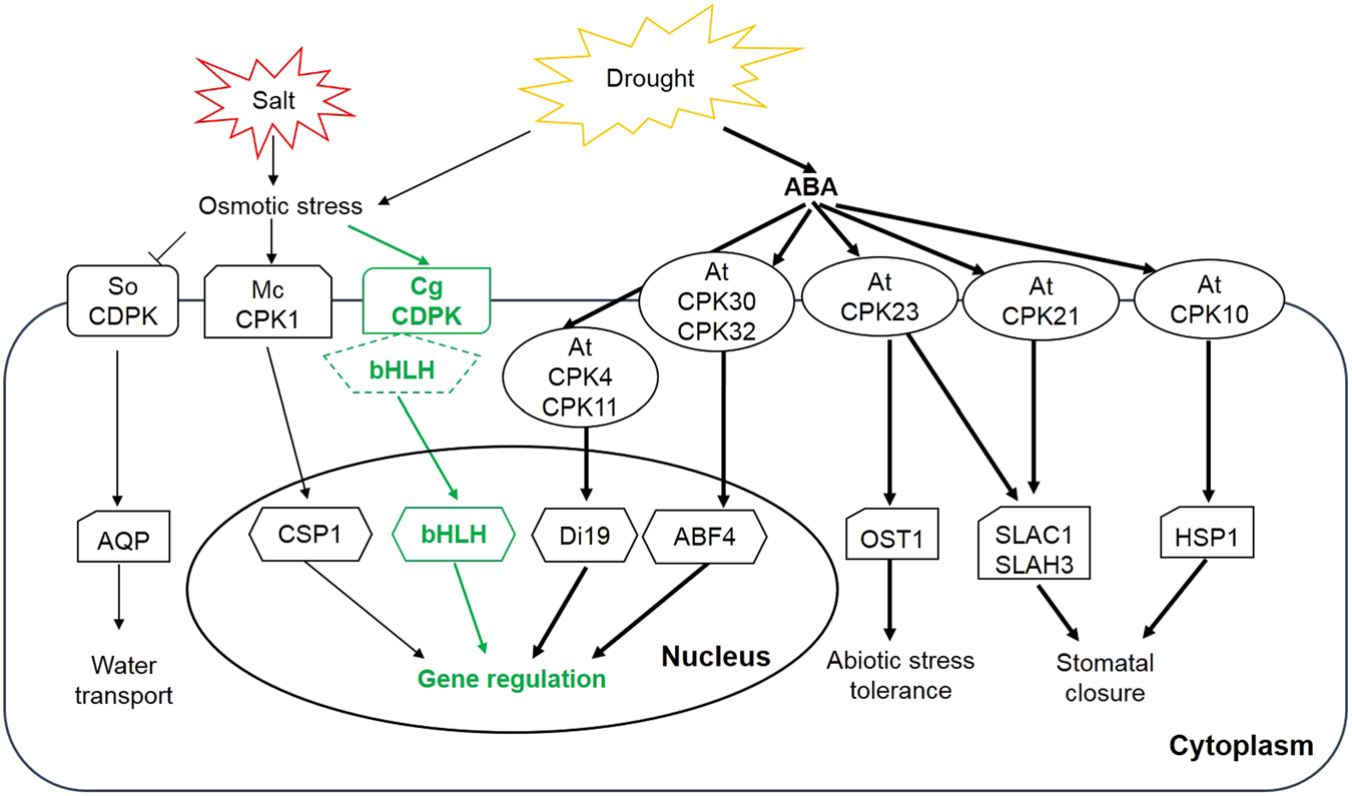
A proposed model of interaction between CgCDPK and CgbHLH001 in CDPK signaling pathway in response to abiotic stresses. The reported interactions between CDPKs and transcription factors or related proteins are shown in solid lines; the suggested interaction between CgCDPK and CgbHLH001 is present in green dashed or solid line. Salt and drought trigger the osmotic stress and ABA synthesis to activate CDPKs, which in turn activate other TFs or function proteins and finally regulate gene expression, water transport, stomatal closure or abiotic stress tolerance. The homologs in Arabidopsis (shorted as At), *Mesembryanthemum crystallinum* (Mc), *Spinacia oleracea* (So) and *Chenopodium glaucum* (Cg) are shown.

In conclusion, we showed that a potential interaction existed between CgCDPK and CgbHLH001 under stress conditions, which was supported by *in vitro* pulldown and *in vivo* BiFC assays, active expression patterns of *CgCDPK* and *CgbHLH001* under stress, and the behavior of *NtbHLH* (a homolog of *CgbHLH001*) in *CgCDPK*-overexpressed transgenic tobacco line. Based on our data, a possible model for the interaction between CgCDPK and CgbHLH001 in response to salt and drought stress is proposed (Fig. 10). Plant may sense salt and drought through osmotic stress (or ABA signaling) to activate CgCDPK, which may in turn interact with CgbHLH001 (or other TFs) on the PM, and finally might enter the nucleus to regulate the target gene expression.

## Methods

### Plant growth and treatments

Surface-sterilized *C. glaucum* seeds were sown in pots containing a 3:1 mixture of vermiculite: perlite (v/v) in the growth chamber under conditions of a 16 h light/8 h dark photoperiod, 25 °C, 10%-20% relative humidity. Plants were cultivated for 4–6 weeks (well-watered and supplying with Hoagland solution at an interval of 2–3 weeks) before next use.

For quantitative RT-PCR (qPCR) analysis of *CgCDPK* and *CgbHLH001* under stress conditions in *C. glaucum*, the above plants were treated with Hoagland solutions containing 50, 100, 300 mmol/L NaCl or 0, 5, 10, 20% polyethylene glycol (PEG) 6000 (mimic drought stress), and sampled at 0, 0.5, 1, 2, 5, 12, 24, 48 h, Hoagland solution only was used as control. For construction of cDNA library of *C. glaucum* under NaCl treatment, 4–6 week-old plants were treated with 300 mmol/L NaCl and sampled at 0, 0.5, 1, 2, 5, 12, 24, 48 h, respectively, then pooled all samples for next use.

For qPCR analysis of *NtbHLH* expression in *CgCDPK*-overexpressed transgenic tobacco line (OE), seeds of non-transgenic (NT) and OE line were surface-sterilized and sown on MS medium till four leaves were present, then plants were transferred into vermiculite: perlite (3:1, *V/V*) mixture and treated with 200 mmol/L NaCl or 15% PEG6000, then sampled at 0, 2, 5, 24 h. For assays, three samples (biological replicates) were collected from young fresh leaves in the upper part of the plant, and immediately frozen in liquid nitrogen until use.

For seed germination test of *CgCDPK*-overexpressed transgenic tobacco lines, four replicates with 30 surface-sterilized seeds of each from three transgenic lines and NT plants were sown on MS medium supplemented with different concentrations of NaCl (0, 50, 100, 200 mmol/L) or PEG6000 (0, 5, 10, 20%). Seed germination was under conditions of a 16 h light/8 h dark photoperiod, 25 °C, 10%-20% relative humidity. Germination was recorded every 24 h for 2 weeks, and then the final germination percentage was calculated. Seed germination experiment was repeated for at least four times to achieve similar results.

### Transcription analysis of *CgCDPK* and *CgbHLH001* genes

Total RNA was isolated from the fresh leaves of *C. glaucum* or tobacco plants using TRIzol Plus RNA Purification Kit (Cat. 15596-026; Invitrogen, USA). First-strand cDNA was synthesized by using the M-MLV reverse transcriptase kit (TaKaRa, Dalian, China) according to the manufacturer’s instructions.

For analysis of the expression pattern of *CgCDPK* and *CgbHLH001* in *C. glaucum*, or *NtbHLH* in *CgCDPK-*overexpressed tobacco transgenic line, the specific primers of each gene were used in qPCR (Table 1). The relative amplification of *β-actin* of *C. glaucum* or *β-actin* of tobacco was used for normalization. The above amplification was performed in the following conditions: 95 °C 2 min followed by 40 cycles of 95 °C 5 s, 60 °C 30 s. qPCR was performed with QuantiNova SYBR Green PCR Kit (Cat. 208054; Qiagen, Germany) and ABI 7500 Real time PCR system (Applied Biosystem, USA). Relative quantification of specific mRNA level was calculated using the cycle threshold (Ct) 2^−∆∆Ct^ method^63^. Four samples (biological replicate) of each treatment were duplicated (technical replicate) in qPCR experiment. The final value of relative quantification was described as fold change of gene expression in the test sample compared to control.

**Table 1:** Primers used in the present study.

Gene	Primer sequence 5′-3′	
	Forward	Reverse
*CgCDPK* (ORF)	CG**GAATTC**ATGGGTATTTGTGCAAGC	ACGC**GTCGAC**TTAAAAGAGTTTAGCAGC
*CgCDPK-GFP* (ORF)	C**GAGCTC**ATGGGTATTTGTGCAAGC	GA**GTCGAC**TCACACGTGGTGGTG
*CgCDPK* (qPCR)	CGAATCACTGCTGCTCAAG	GCGAGTTGCTTGAGCTTG
*CgbHLH001* (ORF)	CG**GAATTC**ATGGATCCACAGACGAC	CC**CTCGAG**TCATGTTTGTCTGTCAAAAC
*CgbHLH001-GFP* (ORF)	C**GAGCTC**GATGGATCCAC	GA**GTCGAC**TCACACGTGGTGGTG
*CgbHLH001* (qPCR)	TCATGTTCGAGCGAGGAGAGG	CGGGGACAAGATCTTGGAGTATTC
*NtbHLH* (qPCR)	TGAGTGCTGAAGAGGGATGAGATACTC	CGAAACTCAACTTGACGCTGCAATG
*Cgactin* (qPCR)	CCTTATTCCATTCCCCAGGCTTC	CATCTGCTCATCAACCTCCTTTGTGC
*Ntactin* (qPCR)	TGGCTCAGAGAGGTTCAGATGTC	CCACCACTAAGGACGATGTTTCC
*CgCDPK-nYFP*	CGGGGTACCATGGGTATTTGTGCAAGC	TTGGGCCCTAAAGAGTTTAGCAGCTGG
*CgbHLH001-cYFP*	CGGGGTACCATGGATCCACAGACGACG	CGGTCGACTCATGTTTGTCTGTCAAAACC
*CgbHLH001-nYFP*	CGGGGTACCATGGATCCACAGACGACG	TTGGGCCCCTGTTTGTCTGTCAAAACC
*CgCDPK-cYFP*	CGGGGTACCATGGGTATTTGTGCAAGC	GCGTCGACTTAAAAGAGTTTAGCAGCTGGATTCTGTGTACC

Note: Letters in bold of the primer indicate restriction endonuclease site.

### Analysis of phylogenetic relationship

The phylogenetic relationship of CDPK or bHLH transcription factors among various plant species was analyzed by using protein sequences from *C. glaucum*, *Arabidopsis thaliana*, *Glycine max*, *Zea mays*, *Oryza sativa*, etc. Multiple alignments were conducted with full-length amino acid sequence by Clustal *W* tool of MEGA 6.06. The phylogenetic tree was constructed through neighbor-joining (NJ) method of MEGA 6.06. Molecular distance of the aligned sequences was calculated according to the *p*-distance parameter, the gaps and missing data were treated as pairwise deletions. Branch points were tested by bootstrap with 1000 replicates. All amino acid sequences used in the present study were acquired from GenBank and Phytozome database.

### Observation of subcellular localization

#### Generation of transgenic tobacco lines with GFP-overexpression

The 3′-end of cDNA of the open reading frame (ORF) of *CgCDPK* or *CgbHLH001* (with the stop codon deletion) was fused to the 5′-end of the green fluorescent protein (GFP) ORF (in-frame), and then inserted into the plant binary expression vector pCAMBIA2300, which resulted in a construct as *35S::CgCDPK-GFP* or *35S::CgbHLH001-GFP* (using construct *35S::GFP* as control). The recombinant vector was transformed into *Agrobacterium tumefaciens* strain EHA105. Leaves from 5–6 week-old tobacco (*N. tabacum*; NC89) were transformed with the recombinant *A. tumefaciens* strain via the leaf disk method^64^ to generate the transgenic tobacco lines.

#### Inspection of GFP signal under confocal microscope

To visualize the subcellular localization of CgCDPK or CgbHLH001, seeds of transgenic tobacco lines (*35S::CgCDPK-GFP* or *35S::CgbHLH001-GFP*) were surface-sterilized and sown on MS medium containing 300 mg/L Kanamycin for two weeks, the survival seedlings were subjected to PCR identification, and the small fresh leaves of PCR positive seedlings (T1) were chose to treat with PM fluorescent marker - DiI (1,1′-dioctadecyl-3,3,3′,3′-tetramethylindocarbocyanine perchlorate) (Solarbio, Shanghai, China). The seedling leaves were gently scratched with scalpel and then stained in working solution (stock solution was prepared in ethanol at 5 mmol/L, and diluted into 10 µmol/L with PBS buffer for ready staining) of PM marker for 15 min at 37 °C. After washing for three times with distilled water, the leaves were inspected with fluorescent signal under confocal microscope (Zeiss LSM 800, Carl Zeiss, Jena, Germany). Seedlings with *35S::GFP* was used as the positive control. All images were visualized and acquired with the ZEN Imaging Software (Version 2.3).

### Assay of stress tolerance of recombinant pET-28a-*CgbHLH001* in *E. coli*

The recombinant strain (Transetta: pET-28a-*CgbHLH001*) and control strain (Transetta: pET-28a) were inoculated in 20 mL fresh LB medium with 50 mg/L kanamycin in 100 mL conical flask containing 0, 200, 400, 600, 800 mmol/L NaCl, or 0, 5, 10, 15, 20% PEG6000 (for drought stress), or 0, 25, 50, 75 µmol/L methyl viologen (MV, for oxidative stress). For test of acid/base tolerance, the culture was adjusted with the pH value at 3.0, 5.0, 7.0, 9.0 or 11.0; for cold tolerance test, the culture was placed at −20 °C for 1, 3, 5 or 7 days and then recovered at 37 °C. All cultures were shaken at 220 rpm for about 4 h till the OD_600_ value reached to 0.5 at 37 °C, with addition of 0.8 mmol/L IPTG, the cultures were cultivated for another 12 h, and then harvested. For measurement of the time course of the recombinant strain growth under 400 mmol/L NaCl, 10% PEG, recovery from −20 °C, 75 µmol/L MV or pH 9.0, 200 μL of cultures were sampled at every 2 h and a total of 12 h was measured. The optical density was determined using spectrophotometer (Benchmark Plus, BIO-RAD, USA).

### Split-ubiquitin based membrane yeast two-hybrid analysis

#### Construction of cDNA library of *C. glaucum* under 300 mmol/L NaCl treatment

First and double-strand (ds) cDNA were synthesized according to the protocol of EasyClone cDNA Library Construction Kit (Cat. P01010; Dualsystem Biotech, Switzerland). dscDNA synthesis was visualized by 1% agarose gel and then completely digested with *Sfi* I restriction endonuclease, cDNA fragments longer than 1000 bp were recovered from a low melting agarose gel, which were then ligated into *Sfi* I digested pPR3-N yeast expression vector, and the ligation mixture was transformed into the competent cells of *E. coli* by chemical method^65^. For CFU (colony forming unit) calculation of the library, 10 µL of the transformed cell culture (with brief cultivation) were diluted 100 times and spread on LB medium with ampicillin and cultivated overnight at 37 °C. When the colonies became clearly visible, CFU was calculated as follows: colonies/10 µL medium × 100 times × total volume of the library (µL). For recombination frequency calculation of the library, 20 randomly selected colonies were examined with the inserted fragments by PCR amplification (using the primers of pPR3-N and pPR3-C) and subjected to agarose gel electrophoresis, the size distribution of inserted fragments was evaluated and the recombination frequency of the library was calculated as follows: colonies with inserted fragments/randomly selected colonies number × 100%.

#### Detection of interaction between bait and preys

The DUALmembrane starter kit SUC (Cat. P01301-P01329; Dualsystems Biotech, Switzerland), a split-ubiquitin based membrane yeast two-hybrid (MYTH) system, was employed in screening the interaction components. For bait-vector construction, the coding sequence of *CgCDPK* containing *Sfi* I restriction sites on both ends was cloned into the pBT3-SUC yeast expression vector to yield pBT3-SUC-*CgCDPK* plasmid in which *CgCDPK* bait gene was fused to the ORF of *Cub-LexA-VP16*. To verify the expression and self-activation ability of bait on the reporter genes, the bait plasmid and the positive pNubG-Fe65 or negative pPR3-N control preys were co-transfected into the NMY32 yeast strain and cultured successively on the dual, triple and quadruple synthetic dropout nutrient medium (SD/-Trp-Leu, SD/-Trp-Leu-His and SD/-Trp-Leu-His-Ade; shorted as SD/-TL, SD/-TLH, SD/-TLHA). cDNA library of *C. glaucum* under 300 mmol/L NaCl treatment was constructed with pPR3-N yeast expression vector as above. pPR3-N-*CgcDNA* plasmid (25 μg) was transformed into the NMY32 yeast strain containing pBT3-SUC-*CgCDPK*, which was then screened on SD/-TLH medium with 5 mmol/L 3-amino-1,2,4-triazole (3-AT), a histidine analog and competitive inhibitor of the *His3* gene product. The positive colonies were further screened by cultivating on SD-TLHA/X-α-gal medium, and measured with β-galactosidase activity (HTX β-galactosidase assay kit, Cat. P01002; Dualsystems Biotech, Switzerland) to test the expression of the reporter gene *LacZ*. The blue colonies were identified and the prey plasmids in which were then re-transfected into NMY32 yeast strain containing pBT3-SUC-*CgCDPK* plasmid for a second-round screening on SD-TLHA/X-α-gal medium. The yield positive colonies were analyzed with the inserted cDNA sequences, which were then compared to GenBank database by using BLAST program available in the NCBI (National Center for Biotechnology Information) and analyzed with the possible functions.

### Expression and purification of CgCDPK or CgbHLH001 protein

Prokaryotic expression vector pGEX-4T-1 (GST-tagged) or pET-28a (His-tagged) was employed in generating recombinant construct pGEX-4T-1-*CgCDPK* or pET-28a-*CgbHLH001*, which was then transformed into *E. coli* BL21(DE3) competent cells. For induction of protein expression, cell culture (0.4–0.6 OD_600_) was treated with 0.5 mmol/L IPTG for 4 h at 37 °C. The target protein was resolved by SDS-PAGE and subjected to Western blot analysis according to Cheng *et al*.^66^ with minor modification: recombinant protein was resolved on 12% SDS-PAGE; the first antibody was the mouse anti-His or anti-GST monoclonal antibody (1:5000 dilution), and then incubated with 1:10000 diluted goat anti-mouse IgG secondary antibody. Both pGEX-4T-1-*CgCDPK* and pET-28a-*CgbHLH001* were partially expressed in the soluble protein of the cell lysate.

For protein purification, cell culture of the recombinant *E. coli* strain was centrifuged at 1,2000 rpm, 4 °C for 10 min, and the deposit was resuspended with phosphate buffered saline (PBS, pH 8.0) and centrifuged for three times, then added 5 mL PBS buffer and incubated at room temperature for 15 min to lyse the cells according to Recombinant Protein Purification Handbook^67^. The cell lysate was further sonicated for 10 min (5 s with 5 s gap at 300 W power) on ice. After centrifugation at 1,2000 rpm, 4 °C for 10 min, soluble protein in supernatant was recovered. For purification of CgCDPK (GST-tagged), the soluble protein was incubated with Pierce™ Glutathione Agarose (Thermo Scientific, USA) for 2 h at 4 °C, after washing with PBS buffer for three times, it was eluted with buffer containing 50 mmol/L Tris, 10 mmol/L reduced glutathione, pH 8.0 for two times. For purification of CgbHLH001 (His-tagged), the soluble protein was incubated with Ni-NTA Agarose (Qiagen, Germany) for 1.5 h at 4 °C and then washed with buffer containing 20 mmol/L Tris-Cl pH8.0, 0.5 mol/L NaCl, 2% Triton X-100, 2 mmol/L β-Mercaptoethanol, 1 mmol/L PMSF, in addition to 10 or 100 mmol/L imidazole for two times of each (the column of this stage could be saved for use in GST-pulldown analysis). Then the Ni column was eluted with the above buffer in addition to 200 mmol/L imidazole for two times. Finally, the above two purified recombinant proteins were checked by SDS-PAGE and Western blot analysis.

### GST-pulldown analysis

The Ni column combined with His-CgbHLH001 saved from purification of His-CgbHLH001 step was used in this experiment. Before pulldown assay, the reduced glutathione was removed from previously purified protein GST-CgCDPK sample by dialysis against TBS buffer (10 mmol/L Tris, 150 mmol/L NaCl, pH 8.0), then the Ni-His-CgbHLH001 resin was incubated with 4 mL purified GST-CgCDPK at 4 °C for 8 h according to the procedure of Pierce^TM^ GST Protein Interaction Pull Down Kit (Cat. 21516; Thermo Scientific, USA). After incubation, the resin was washed three times at 18 rotate/min with 4 mL PBS buffer (pH 8.0) for 5 min of each. Finally, proteins were eluted two times with 200 mmol/L and two times with 500 mmol/L imidazole in 1 mL buffer containing 20 mmol/L Tris-Cl pH 8.0, 0.5 mol/L NaCl, 2% Triton X-100, 2 mmol/L β-Mercaptoethanol, 1 mmol/L PMSF, a total of 4 mL eluate was pooled and then resolved by 12% SDS-PAGE and analyzed by Western blot (according to the method described above).

### Bimolecular fluorescence complementation (BiFC) assay

#### Constructs for BiFC assay

Full-length coding sequences of *CgCDPK* and *CgbHLH001* were cloned into the binary nYFP and/or cYFP vectors, respectively, to generate four constructs: *35S::CgCDPK-nYFP*, *35S::CgbHLH001-nYFP*, *35S::CgCDPK-cYFP*, and *35S::CgbHLH001-cYFP* according to previously described protocols^68^. Primers used for vector construction were present in Table 1. These constructs were transformed into *Agrobacterium tumefaciens* GV3101 strain through CaCl_2_ transformation^69^.

#### Detection of interactions *in vivo*

Recombinant *A. tumefaciens* strains (GV3101) containing different constructs were incubated, harvested and resuspended in infiltration buffer (10 mmol/L MES, 0.2 mmol/L acetosyringone and 10 mmol/L MgCl_2_) at a final concentration of OD_600_ = 0.4, then allowed to stand at room temperature for 1–3 h. Equal amounts of the *Agrobacterium* suspension of each construct were mixed into a new 1.5 mL tube and vortexed for 10 sec to be ready for use. Five- to six-week-old *N. benthamiana* plants were prepared for infiltration. Placed the tip end of the syringe (without needle) against the underside of the leaf (avoiding the veins) by supporting with one finger on the upperside, then gently pressed the syringe to infiltrate the *Agrobacterium* mixture into the fresh leaf, followed by infiltration of DiI fluorescent PM marker (10 µmol/L working solution) nearby and labeled the infiltration area for future recognition. Treated plants were kept in darkness overnight, and then transferred to normal growth conditions for 48 h. The YFP and DiI fluorescent signals in the leaf of *N. benthamiana* were examined under the confocal microscope (Zeiss LSM 800, Jena, Germany) with excitation at 514 and 549 nm, respectively.

### Statistical analysis

All data were analyzed by Microsoft Excel 2010 and GraphPad Prism 5.0 (GraphPad Software, San Diego, USA). For qPCR, three biological replicates with two technical replicates of each treatment were measured. For seed germination, four biological replicates with 30 seeds of each were applied. For *E. coli* stress treatment, three biological replicates of each treatment were assayed. Two-way ANOVA was used to test the significance of main effects. Differences were measured by Post Hoc Duncan multiple comparison test at 0.05, 0.01 or 0.001 significance level.

## Electronic supplementary material

Supplementary Figure 1
