# Author Correction: The bHLH transcription factor CgbHLH001 is a potential interaction partner of CDPK in halophyte *Chenopodium glaucum*

**DOI:** 10.1038/s41598-018-37592-6

**Published:** 2018-12-21

**Authors:** Juan Wang, Gang Cheng, Cui Wang, Zhuanzhuan He, Xinxin Lan, Shiyue Zhang, Haiyan Lan

**Affiliations:** 1 Xinjiang Key Laboratory of Biological Resources and Genetic Engineering, College of Life Science and Technology, Xinjiang University, Urumqi, 830046 China; 2 Institute of Economic Crops, Xinjiang Academy of Agricultural Sciences, Urumqi, 830091 China

Correction to: *Scientific Reports* 10.1038/s41598-017-06706-x, published online 16 August 2017

This Article contains errors in the Results section, under the subheading ‘Analysis of interaction between CDPK and bHLH in *C. glaucum*’,

“The three top ranking models of the CgCDPK-CgbHLH001 interaction complex were presented in Fig. 5c”

should read:

“The three top ranking models of the CgCDPK-CgbHLH001 interaction complex were presented in Fig. 6c”

In addition,

“Prediction of phosphorylation sites of CgbHLH001 revealed 39 potential amino acid residues (Fig. 6d), in which the motif of ^61^-GKRLKS-^66^ was a putative action site for CgCDPK.”

should read:

“Prediction of phosphorylation sites of CgbHLH001 revealed 39 potential amino acid residues (Fig. 6d), in which the motif of ^91^-GKRLKS-^96^ was a putative action site for CgCDPK.”

