# Supplementary material for: The bHLH transcription factor CgbHLH001 is a potential interaction partner of CDPK in halophyte Chenopodium glaucum
Source: Sci Rep. 2017 Aug 16;7:8441. doi: 10.1038/s41598-017-06706-x (PMC5559460; doi:10.1038/s41598-017-06706-x)
Supplement: Supplementary file 1 — Supplementary Figure 1 [file 41598_2017_6706_MOESM1_ESM.pdf]

The bHLH transcription factor CgbHLH001 is a potential interacting partner of CDPK in halophyte *Chenopodium glaucum*

Juan Wang[1,2]*, Gang Cheng[1]*, Cui Wang[1], Zhuanzhuan He[1], Xinxin Lan[1], Shiyue Zhang[1], Haiyan Lan[1]**

1. Xinjiang Key Laboratory of Biological Resources and Genetic Engineering, College of Life Science and Technology, Xinjiang University, Urumqi 830046, China
2. Institute of Economic Crops, Xinjiang Academy of Agricultural Sciences, Urumqi 830091, China

* These authors contributed equally to this work.

** Corresponding author. E-mail address: lanhaiyan@xju.edu.cn

Running title: Analysis of interaction between CDPK and bHLH in *Chenopodium glaucum*

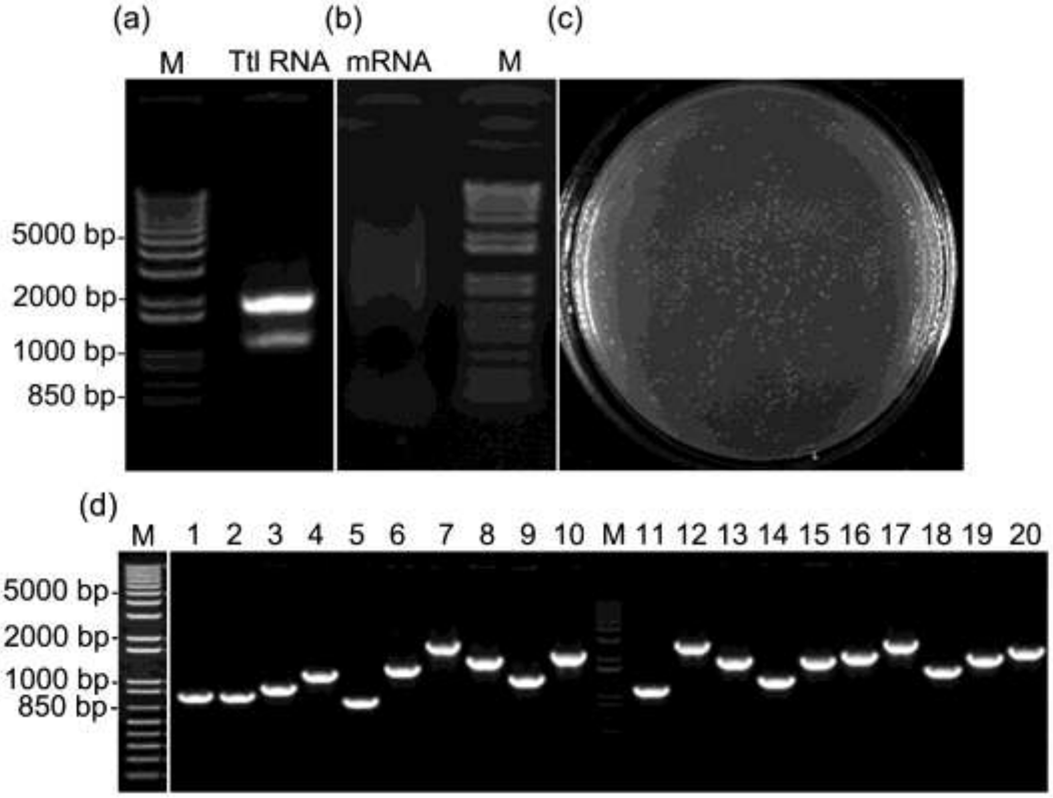

Supplementary Fig. 1 Total RNA isolation **(a)**, mRNA purification **(b)**, cDNA library construction **(c)** and recombinant frequency detection **(d)**. M: DL12,000 marker. In (a), Ttl RNA: Total RNA of *C. glaucum* leaf. In (b), mRNA: messenger RNA. In (c), 1-20: recombinant colonies.
